# Intraluminal Thrombus Characteristics in AAA Patients: Non-Invasive Diagnosis Using CFD

**DOI:** 10.3390/bioengineering10050540

**Published:** 2023-04-27

**Authors:** Djelloul Belkacemi, Miloud Tahar Abbes, Mohammad Al-Rawi, Ahmed M. Al-Jumaily, Sofiane Bachene, Boualem Laribi

**Affiliations:** 1 Mechanics and Energetics Laboratory, Hassiba Ben Bouali University, Chlef 02000, Algeria; belkacemidjelloul@gmail.com (D.B.);; 2 Unité de Développement des Equipements Solaires UDES, CDER, Bousmail, Tipaza 42415, Algeria; 3 Center for Engineering and Industrial Design, Waikato Institute of Technology, Hamilton 3240, New Zealand; mohammad.al-rawi@wintec.ac.nz; 4 Institute of Biomedical Technologies, Auckland University of Technology, Auckland 1010, New Zealand; 5 Radiologie, Centre d’Imagerie Médicale, Cheraga, Algiers 16000, Algeria; 6 FIMA Laboratory, Department of Technology, Djilali Bounaama University, Khemis Miliana 44225, Algeria

**Keywords:** abdominal aortic aneurysm, intraluminal thrombus thickness, computational fluid dynamics, non-Newtonian model, wall shear stress based parameters

## Abstract

Abdominal aortic aneurysms (AAA) continue to pose a high mortality risk despite advances in medical imaging and surgery. Intraluminal thrombus (ILT) is detected in most AAAs and may critically impact their development. Therefore, understanding ILT deposition and growth is of practical importance. To assist in managing these patients, the scientific community has been researching the relationship between intraluminal thrombus (ILT) and hemodynamic parameters wall shear stress (WSS) derivatives. This study analyzed three patient-specific AAA models reconstructed from CT scans using computational fluid dynamics (CFD) simulations and a pulsatile non-Newtonian blood flow model. The co-localization and relationship between WSS-based hemodynamic parameters and ILT deposition were examined. The results show that ILT tends to occur in regions of low velocity and time-averaged WSS (TAWSS) and high oscillation shear index (OSI), endothelial cell activation potential (ECAP), and relative residence time (RRT) values. ILT deposition areas were found in regions of low TAWSS and high OSI independently of the nature of flow near the wall characterized by transversal WSS (TransWSS). A new approach is suggested which is based on the estimation of CFD-based WSS indices specifically in the thinnest and thickest ILT areas of AAA patients; this approach is promising and supports the effectiveness of CFD as a decision-making tool for clinicians. Further research with a larger patient cohort and follow-up data are needed to confirm these findings.

## 1. Introduction

Abdominal aortic aneurysm (AAA) is one of the most common cardiovascular diseases (CD) accounting for 1.7–5% people over 65 years of age [1] and reaching up to 10% for 80-year-old male subjects [2]. AAA is characterized by localized deformation and dilatation of the abdominal aorta (usually infrarenal) with 1.5 times the diameter of the healthy aorta [3,4]. AAA can undergo sudden rupture, frequently fatal, making it one of the most common causes of death in the United States [5]. It accounts for up to 4–5% of all sudden deaths [6], and it is the 14th most common cause of death in the world [7]. The total number of worldwide deaths reached 172,000 in 2019 including both thoracic and abdominal aneurysms [8].

In clinical practice, the most used criterion to select eligible patients for surgery relies on the measurement of the AAA maximum diameter [9,10]. Recent guidelines continue to recommend a threshold diameter of 5.5 cm as a unique criterion to decide whether the resort to surgery is required; however, some clinicians consider 5.0 cm for women as a threshold [1,11]. Assessing the risk of rupture is extremely important in reducing AAA mortality. Thus, there is a need for additional criteria to help surgeons make a more informed decision regarding the surgical procedure and management of patients with AAA. Several efforts have been devoted for estimating rupture risk. Forneris et al. [12] suggested a new parameter combining time-averaged wall shear stress (TAWSS), intraluminal thrombus (ILT) and wall stress to determine regions of possible rupture. AAA is often associated with deposition of blood clots and cell debris in the ILT as well as breakdown of connective tissue in the wall. This plays an important role in the rupture and the remodeling of the wall, making the detection and the prediction of ILT growth crucial.

Using computational fluid dynamics (CFD), the blood flow can be modeled without requiring invasively obtained or experimental data. Flow features including WSS and its derivatives can be obtained within the CFD model. To understand the mechanism of ILT accumulation in AAAs, several numerical and computational simulations were carried out over the last decade. Chen et al. [13] found a positive relationship between the particle residence time and the platelet activation for ILT. Lozowy et al. [14] studied the effect of jet impingement on ILT deposition in AAA patients and concluded that the jet impingement regions may prevent ILT deposition. Chandra et al. [15] observed the absence of ILT in high WSS areas. Colciago et al. [16], in their study of patients presenting with thin ILT, showed that simulation-based indicators could support clinicians in the assessment of patients with AAA. Zambrano et al. [17] also conducted a follow-up study on a cohort of patients in order to investigate the relationship between TAWSS, ILT growth, and aneurysm expansion. Qui et al. [18] assessed, via computational fluid dynamics simulation, the near-wall hemodynamic parameters in ruptured AAA patients.

Previous studies had originally shown the existence of a correlation between ILT accumulation and low TAWSS [19,20,21]. Another parameter that has been suggested to correlate with ILT deposition is the oscillation shear index (OSI). Some studies have found an association between low OSI and ILT accumulation [22,23]. Other studies contradicted this and found high OSI coincide with regions of ILT [24] while others have demonstrated that ILT was shown to occur at both high and relatively low OSI [14,19]. This contradiction has prompted scientists to seek other parameters related to ILT accumulation. Zambrano et al., 2022 [19] pointed out this inconsistency and suggested to investigate the relationship between vortical structure and ILT.

In this study, a CT scan images-based CFD study is performed on three selected AAA patients. These patients show different patterns of ILT deposition; thus, the relationship between some possible non-invasive assessment hemodynamic indicators and ILT deposition can be investigated. To explore whether monodirectional or multidirectional flow near the wall will affect ILT deposition, the transversal WSS (TransWSS) is studied. In addition to TAWSS and OSI, two parameters—relative residence time (RRT) and the endothelial cell activation potential (ECAP)—which combine TAWSS and OSI are studied. Additionally, based on its thickness, ILT is regionalized to the thickest and thinnest areas with the aim to explore whether it is possible to use this information as an additional indicator to predict ILT growth region based on hemodynamic measures in these regions. This suggested approach could be used to score regions by their degree of ILT growth potential and consequently could be used in AAA rupture risk prediction.

## 2. Materials and Methods

### 2.1. Workflow

**Figure 1:**
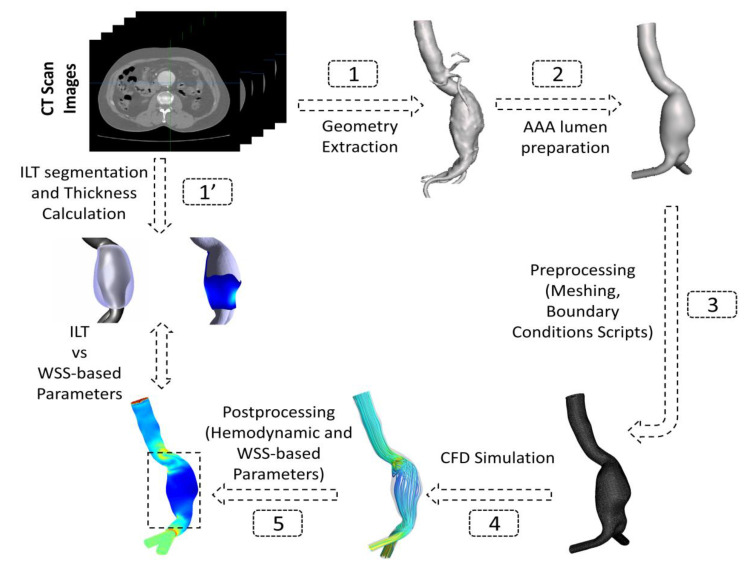
Schematic representation of the adopted workflow showing the steps followed from CT images segmentation to post-processing of CFD-based data.

The patient-specific geometries of 3 male volunteers with a mean age of 68 years diagnosed with AAA were reconstructed from Computed Tomography (CT) scans retrospectively acquired between the 3rd and 17th of February 2021 under ethics approval number (00064). The selected volunteer patients showed different ILT deposition configuration and thickness (Table 1) with the aim to test the methodology adopted in this work on patients with different characteristics. These patients were labeled as P1, P2, and P3 (Table 1). Figure 1 shows a schematic diagram of the process adopted in this study. The geometries were trimmed and prepared to be used in the CFD simulations, and, finally, data post-processing was performed to extract the hemodynamic parameters and WSS derivatives from CFD results. All the steps are detailed in the following sections.

### 2.2. Data Acquisition and Patient-Specific Geometries Reconstruction

The CT scans were performed and provided by a radiologist from a medical imaging center, with a slice thickness of 2 mm for P1 and P2 and of 1 mm for P3. To extract the aneurysm’s lumen wall, SimVascular open-source software was used [25]. The arterial wall was smoothed to reduce roughness by using the shape-preserving smoothing filter and then clipped at the two iliac arteries to create the outlets’ surfaces using Autodesk Meshmixer software (www.meshmixer.com accessed on 22 March 2023). The descending aortas were clipped distally so as not to influence the flow, preserving sufficient inlet lengths which were calculated using an expression from Wood et al., 1999 [26]. Furthermore, both outlets were extruded with lengths of 5d, where d is the average diameter of the iliac arteries.

**Figure 2:**
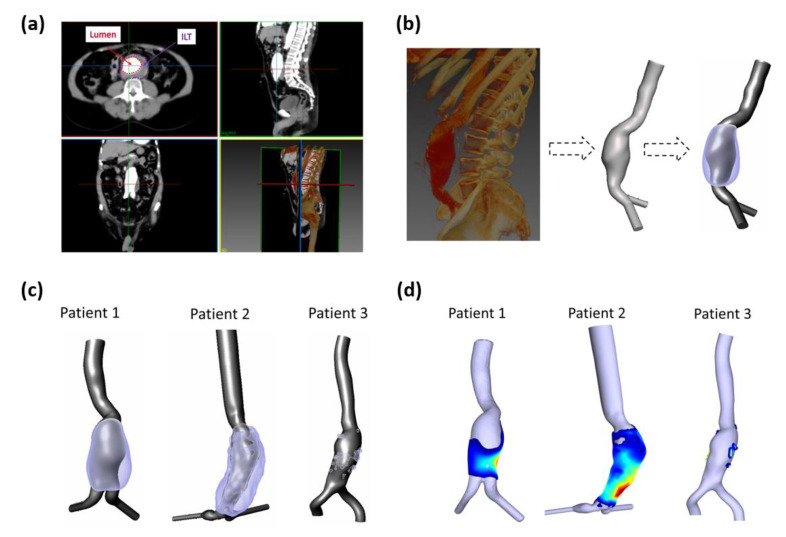
(**a**) Example of CT scan slices in axial, sagittal, coronal, and 3D rendered views; lumen and ILT regions are shown in axial views; (**b**) Rendered left sagittal image and segmented artery with ILT of AAA for P1. (**c**) Extracted computational domains of three AAA patients-specific lumens used to perform numerical simulations (grey) and ILT regions (purple); (**d**) Calculated ILT thickness patched to the lumen surface where blue corresponds to low values (thin) and red to high values (thick).

ILT wall segmentation was also obtained using SimVascular (Figure 2c). To estimate the ILT thickness, the coordinates of (i) the outer ILT surface and (ii) the surface of the aortic lumen for the three aneurysm models were exported to a purposely developed MATLAB script (Figure 2d). The distance between the two surfaces is calculated using the minimum Euclidean distance for each point of the two surfaces. In vivo studies have estimated the AAA wall thickness to vary between 1 mm and 4.26 mm [27,28]. In our work, it is assumed to be constant with 2 mm of thickness. To obtain the final ILT thickness, a mask of 2 mm was applied to subtract the wall thickness from the initially estimated ILT thickness. For visualization purposes and to be compared against WSS-based parameters, the final ILT thickness values were patched to the aortic lumen surface (Figure 2d). The final obtained geometries are displayed in Figure 2c,d. More details about the three AAAs are reported in Table 1.

### 2.3. Patient Specific CFD Simulation

**Figure A2:**
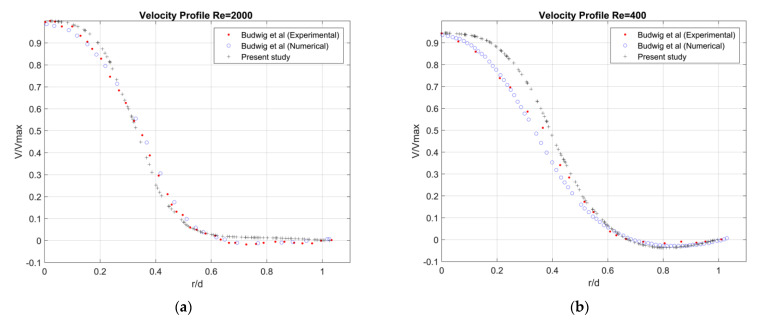
Comparison of steady profile across the center of the AAA (D/d = 2.1, L/d = 8) (**a**) Re = 2000, (**b**) Re = 400.

The patients’ geometries, which were previously segmented in SimVascular from the CT images, were subsequently imported and discretized into a volumetric mesh of tetrahedral elements using ANSYS ICEMCFD (ANSYS, Inc., Canonsburg, WA, USA). Mesh sensitivity analysis was performed with the aim of defining the optimum computational domain for each AAA model. Unsteady simulations were conducted for each patient using three different mesh resolutions: Coarse, Medium, and Fine. The comparison was performed at the systolic peak by calculating the Grid Convergence Index (GCI). GCI is widely recognized as the most reliable method for quantifying numerical uncertainty [29]. For more details about mesh sensitivity analysis see Appendix A. Near the wall, an exponential growth ratio was adopted with a minimum element size of 0.2 mm and a total thickness of approximately 2.6 mm. Details of the final meshes are described in Table 2. Four cardiac cycles were simulated to minimize the influence of initial conditions, and the last cycle was considered in our results, as the results become asymptotic after the third cycle; consequently, more cycles are not required [17]. The aortic wall was assumed to be rigid with no-slip condition [30,31,32,33,34,35]. Note that the arterial wall is commonly treated as rigid for elderly AAA patients as a small change in wall deformation was observed [36]. To validate our numerical model, we compared the axial velocity profile at the center of the aneurysm to the experimental and numerical data reported by Budwig et al. [37] for blood flow through an axisymmetric AAA model (for more details about the validation see Appendix B, Figure A2).

**Figure 3:**
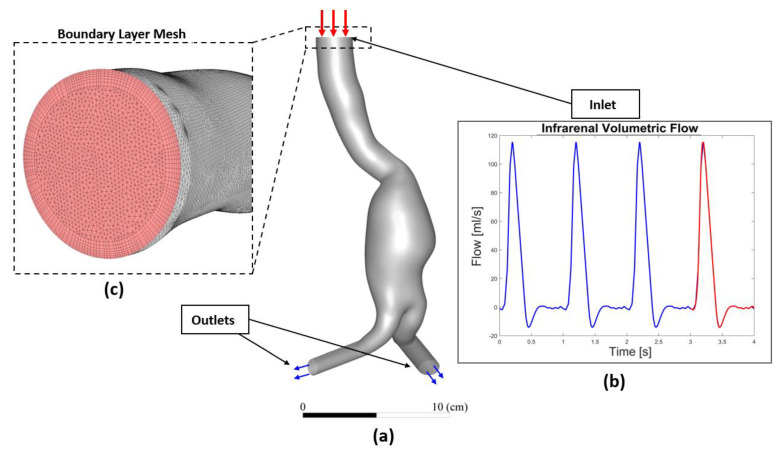
(**a**) Overall 3D AAA model (P1 is shown as an illustration) (**b**) subjected to inflow pulsatile velocity waveform as inlet boundary condition used in the numerical model (the waveform displayed corresponds to flow rate instead of velocity), and (**c**) Boundary layer and topology of the mesh at the inlet face.

Due to the lack of patients’ specific boundary conditions, we decided to use in vivo data from the literature. Using phase-contrast MRI (PC-MRI) data, the flow waveforms at the infrarenal artery were extracted by Les et al. and interpolated from a cohort of 36 AAA patients, and then an averaged volumetric flow was deduced [38]. In our study, this PC-MRI-based volumetric flow was converted to a corresponding velocity waveform and applied as an inlet boundary condition for each patient (an example shown for P1 in Figure 3). A zero-reference pressure was imposed at the outlet boundaries [14,39,40,41]. The temporal variations of the physiological velocity inlet condition were reproduced by Fourier series and implemented through a “user_defined_function” (UDF) script.

### 2.4. Governing Equations

In this study, the flow is modeled as non-Newtonian, and to capture blood’s shear-thinning behavior the Carreau–Yasuda (C–Y) model was used [19,42,43,44,45,46,47]. The C–Y model was implemented using the UDF script for blood viscosity and was expressed as follows:

(1) $$\mu =\mu_{\infty }+\left(\mu_{o}-\mu_{\infty }\right)*{\left(1+{\left(\lambda \dot{\gamma }\right)}^{a}\right)}^{\frac{n-1}{a}}$$

where *η*, $\dot{\gamma },\;\mu_{\infty }$ and $\mu_{o}$ are the viscosity, shear rate, viscosity at infinite shear-rate and the zero-shear viscosity, respectively. *λ*, *α* and *n* are material coefficients (*λ* = 1.902 s, *a* = 1.25, *n* = 0.22). For blood, $\mu_{\infty }$ = 0.00345 Pa s, $\mu_{o}$ = 0.056 Pa s [42,45,46,47]. In the literature, different variables of the C–Y model exist; in this study, we decided on the set of values used in the original paper and used recently by Tzirakis et al. [45].

The flow in the abdominal aorta was hence described by the incompressible Navier–Stokes equations under the assumption of laminar flow ($\mathrm{density}\;\rho =1050\;\mathrm{kg}/m^{3})$:

(2) $$\;\rho \frac{\mathrm{dv}}{\mathrm{dt}}=-\nabla p+\mu \;\Delta v\;$$

(3) ∇· V=0 

In this study, the finite volume method (FVM) was adopted to solve the governing equations and to predict the time-dependent flow through three-dimensional AAA geometries by using Ansys Fluent with the implicit solver. A second-order upwind scheme was used for spatial discretization and the SIMPLE algorithm (Semi-Implicit Method for Pressure Linked Equations) for pressure–velocity coupling. Each pulse cycle was divided into 1200 time steps of 83.333 × 10^−2^ ms. The convergence criteria for the solutions were considered when the residuals for the continuity and the velocity achieved 1 × 10^−5^.

### 2.5. Wall Parameters’ Analysis

Hemodynamic wall parameters including TAWSS, Oscillatory Shear Index (OSI) [48], TransWSS [49], Relative Residence Time (RRT) [50] and ECAP are documented. A custom MATLAB script is used for the post-processing and the calculation of those parameters using the following equations, as we have previously used it [51,52]:

(4) $$\;\mathrm{TAWSS}=\frac{1}{T}\int_{0}^{T}\left|\vec{\mathrm{wss}}\right|dt$$

where $\vec{\mathrm{wss}}$ represents the instantaneous WSS vector; *t* represents the time; and *T* represents the cardiac cycle.

The OSI is a mechanical factor related to flow oscillation throughout the cardiac cycle. It represents the temporal variation in WSS direction which has been shown to affect the endothelial cells’ (EC) behavior. This dimensionless scalar index is defined by

(5) $$\;\;\;\;\;\;\;\;\;\;\;\;\;\;\mathrm{OSI}=\frac{1}{2}\left(1-\frac{\left|\int_{0}^{T}\vec{\mathrm{wss}}\;dt\right|}{\mathrm{TAWSS}}\right)$$

RRT is a mechanical factor which includes the effects of both OSI and the time-averaged WSS magnitude. It is defined as

(6) $$\;\mathrm{RRT}=\frac{1}{\left(1-2\cdot \mathrm{OSI}\right)\cdot \mathrm{TAWSS}}$$

The endothelial cell activation potential (ECAP) [24] characterizes the degree of thrombogenic susceptibility of EC. This parameter localizes regions of high OSI and low TAWSS by using the ratio of OSI and the TAWSS.

(7) $$\mathrm{ECAP}=\frac{\mathrm{OSI}}{\mathrm{TAWSS}}$$

To distinguish between multidirectional and uniaxial flows, transversal WSS (TransWSS) was introduced [49]. TransWSS completes TAWSS and OSI instead of replacing them. This metric is defined as follows:

(8) $$\;\mathrm{TransWSS}=\frac{1}{T}=\frac{1}{T}\int_{0}^{T}\left|\vec{wss}\cdot \left(\vec{n}x\frac{\int_{0}^{T}\vec{wss}\;dt}{\left|\int_{0}^{T}\vec{wss}\;dt\right|}\right)\right|dt$$

where $\vec{n}$ represents the normal to the arterial surface.

## 3. Results

### 3.1. Velocity Field and ILT Deposition

**Figure 4:**
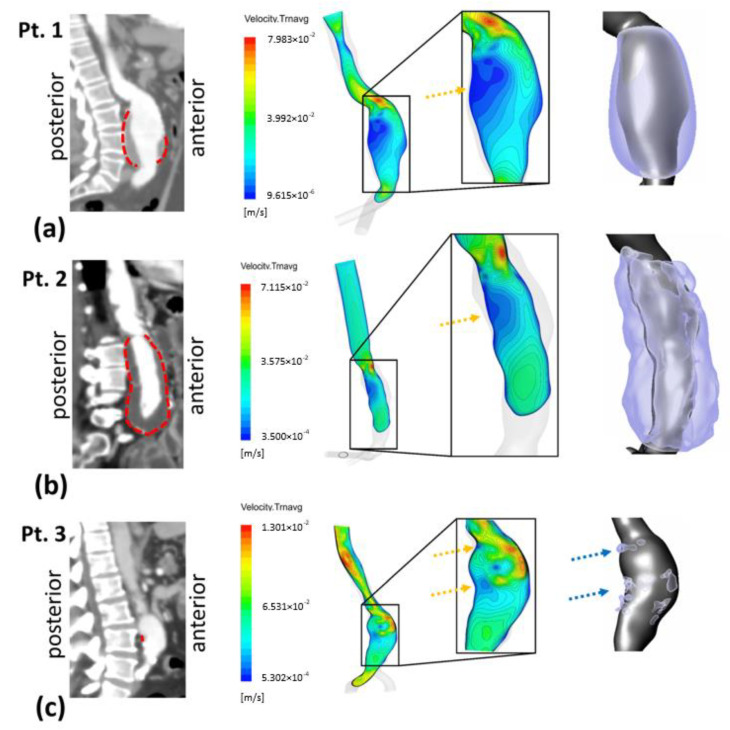
Right sagittal CT images with dashed red lines showing outer ILT walls (left column). Time-averaged velocity contours at right view of mid-sagittal section and magnified in the AAA sac region (middle column); yellow arrows show recirculating flow zones: (**a**) Patient 1 (**b**) Patient 2 and (**c**) Patient 3. Extracted computational domain (grey) and ILT deposition (purple) of AAA patients are shown in the rightmost column; blue arrows indicate ILT deposition areas for Patient 3.

To explore the relationship between hemodynamic parameters and ILT deposition, a qualitative comparison was preliminarily performed by visualizing time-averaged velocity contours. In addition to the mid-sagittal CT image, Figure 4a–c also show the time-averaged velocity contours in the corresponding section for the three patients—P1, P2 and P3, respectively. The ILT deposition zones are delimited by red dashed lines in CT images and as a 3D representation with the AAA computational domain. ILT covers the entire AAA sac in P1 and P2 except the anterior proximal wall for P1, whereas, for P3, small and sparse areas of ILT are detected.

At the inlet of the aneurysms (the proximal neck), a jet is formed and penetrates into the aneurysmal sac. The velocity gradient between the jet flow and the surrounding parts produces shear layers, generating recirculation zones. Time-averaged velocity magnitude contours in the mid-sagittal section show apparent low-velocity magnitude regions along the posterior wall in P1 and P2 (indicated by the yellow arrows). The formed jet is close to the proximal anterior wall for the three patients (Figure 4).

### 3.2. ILT Deposition and WSS Derivatives

**Figure 5:**
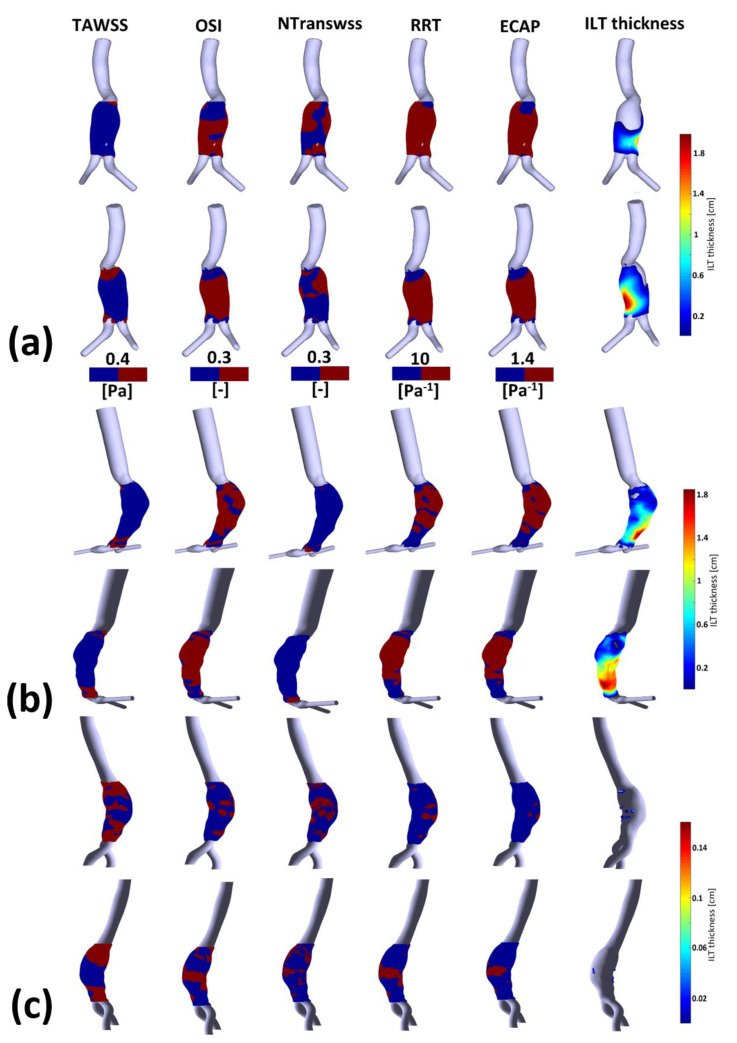
Visualization of TAWSS, OSI, NTransWSS, RRT and ECAP (respectively from left to right columns) in three AAA patients using CFD simulations in the AAA zone with threshold values indicated in the corresponding colormaps (blue for values below threshold and red for values above). In the rightmost column, ILT thickness in [cm] is shown for: (**a**) P1; (**b**) P2; and (**c**) P3. For visualization purposes, two views are displayed for each patient.

To further elucidate the ILT deposition–WSS derivatives’ relationship, Figure 5 shows WSS derivatives thresholds versus ILT thickness. Since the follow-up data of patients were not available and in order to determine the hemodynamic parameters favorable to ILT growth, we used thresholds previously reported in the literature [20,21,33,49,53,54,55,56]. The literature shows different cut-off values of WSS derivatives as key indicators to distinguish regions with low or high potential for thrombus formation. In this work, we decided to use the most common ones. The low TAWSS level in the aorta is defined as a hemodynamic condition favorable for ILT deposition; particularly, <0.4 Pa [20,33,53,54,55], OSI values > 0.2 or >0.3 [21,33,49,54], ECAP > 1.4 Pa^−1^ [21] and values > 10 Pa^−1^ for RRT [33,54] are also considered corresponding to ILT deposition areas. ILT thickness shown in rightmost column was calculated using a MATLAB script (see Section 2.2).

**Figure A3:**
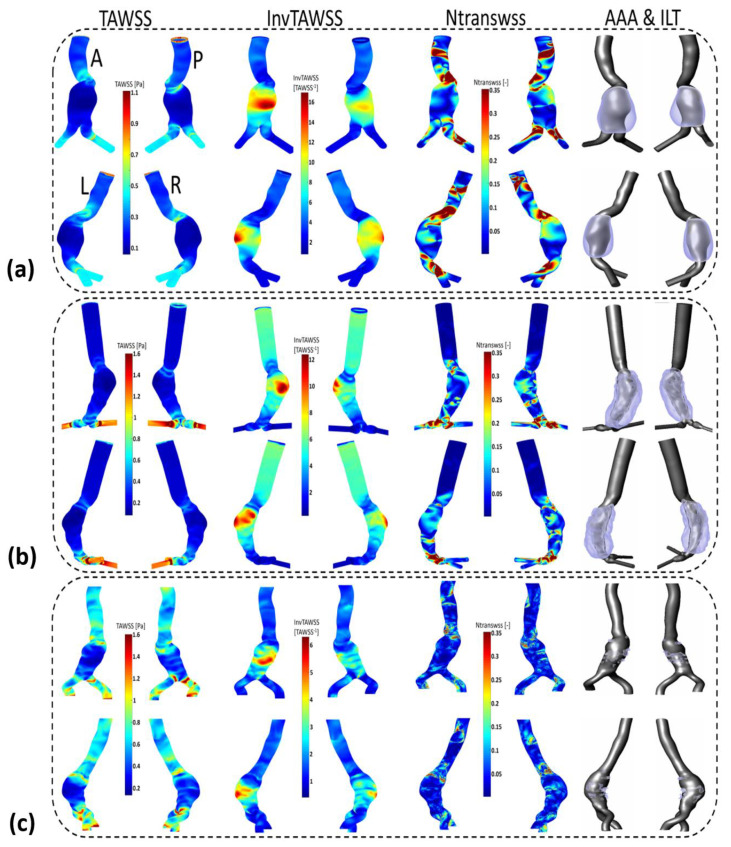
Visualization of TAWSS, InvTAWSS and normalized TransWSS (NTransWSS) distributions (respectively, from left to right columns) in AAA patients using CFD simulations: (**a**) Patient 1 (**b**) Patient 2 and (**c**) Patient 3. Extracted computational domain (grey) and ILT deposition (purple) of AAAs patients derived from CT scan images are shown in the rightmost column as 3D representation. Posterior P, anterior A, left L and right R views are displayed.

**Figure A4:**
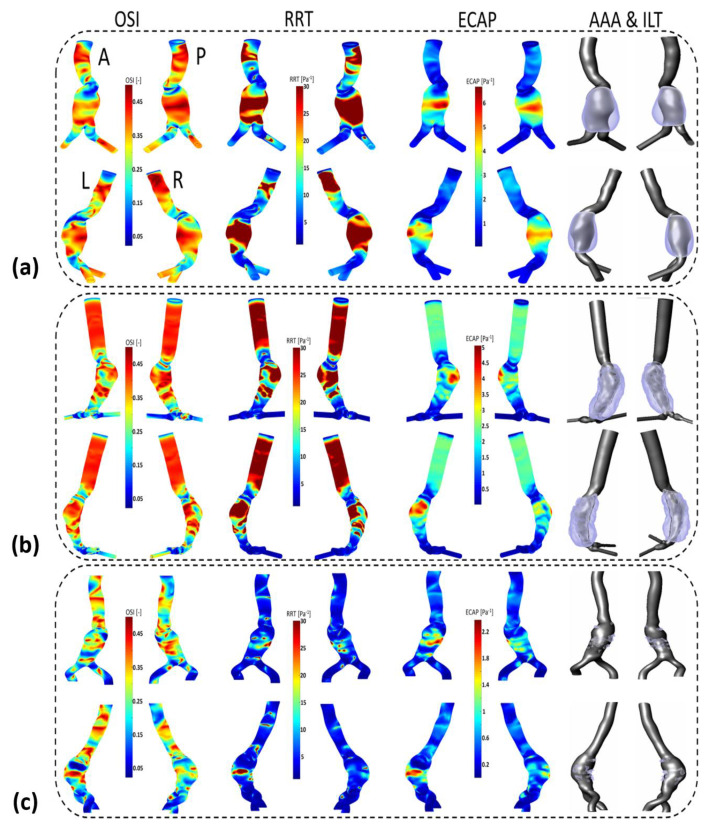
Visualization of OSI, RRT and ECAP (respectively, from left to right columns) in AAA patients using CFD simulations: (**a**) Patient 1 (**b**) Patient 2 (**c**) Patient 3. Extracted computational domain (grey) and ILT deposition (purple) of AAA patients derived from CT scan images are shown in the rightmost column as 3D representation. Posterior P, anterior A, left L and right R views are displayed.

Figure A3 shows the TAWSS, invTAWSS (the inverse of the TAWSS) and normalized TransWSS contours for P1, P2 and P3. TransWSS is normalized with respect to the maximum values of each patient, namely NtransWSS = TransWSS/max(TransWSS). Contours of OSI, RRT and ECAP for P1, P2 and P3 are shown in Figure A4 (for more details, see Appendix C).

**Figure 6:**
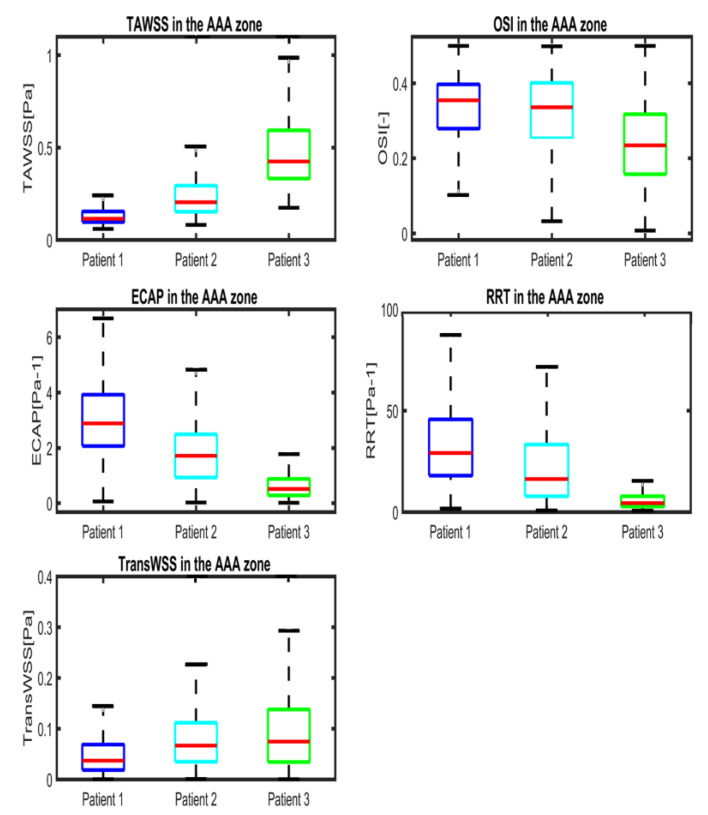
Boxplots of WSS-based parameters (TAWSS, OSI, ECAP, RRT and TransWSS) in the entire AAA region for P1 (Patient 1), P2 (Patient 2) and P3 (Patient 3). Whiskers refer to the 10th and the 90th percentiles, respectively; each box ranges between the first and the third quartiles with red line designating the median value of the WSS derivatives.

WSS derivatives at the aorta (parent vessel) and the iliac arteries are masked, showing only the aneurysmatic region which is the region of interest (ROI). In this region, ILT covers partially the entire AAA’s sac for P1 or covers entirely the sac for P2. However, for P3 (Figure 5c), a small part of the aneurysm sac is covered by ILT near its maximum diameter. TAWSS is low, and OSI is high in almost the ROI (Figure 5). It can be seen from the statistical boxplots (Figure 6) that the medians of TAWSS values are 0.14, 0.241 and 0.48, whereas the medians of OSI values are 0.32, 0.324 and 0.23 for P1, P2, and P3, respectively. ECAP and RRT levels are higher in patients with ILT (P1 and P2) compared to patients characterized by the initiation of ILT (P3).

Table A2, Table A3 and Table A4 (see Appendix D) succinctly summarize the nature of the flow near the wall for all compartments in P1, P2 and P3, respectively. From these three tables, it can be observed that regions of low TAWSS and high OSI are subjected to ILT deposition (as per the rightmost columns in the three tables) for all of the aneurysms regardless of NtransWSS. In these regions (i.e., low TAWSS and high OSI), the EC are subjected to oscillating non-disturbed flow (low TransWSS) or oscillating disturbed flow (high TransWSS) (see TAWSS, OSI and NtransWSS in Figure 5).

One difference between P1 and P2 should be noted; for P2, the thickest ILT area is in the distal part of the aneurysm sac and corresponds to low values of OSI, RRT and ECAP (the blue area in Figure 5b) as well as high values of TAWSS (red area), while in P1, the ILT is also thickest in the distal part but correlates opposite to P2’s hemodynamic conditions in the corresponding zone. For Patient 1, these hemodynamic conditions correspond to the proximal anterior part of the aneurysm sac (entrance) which is devoid of ILT.

### 3.3. ILT-Thickness Regionalization as Possible Indicator of Thrombogenic Prone Regions

**Figure 7:**
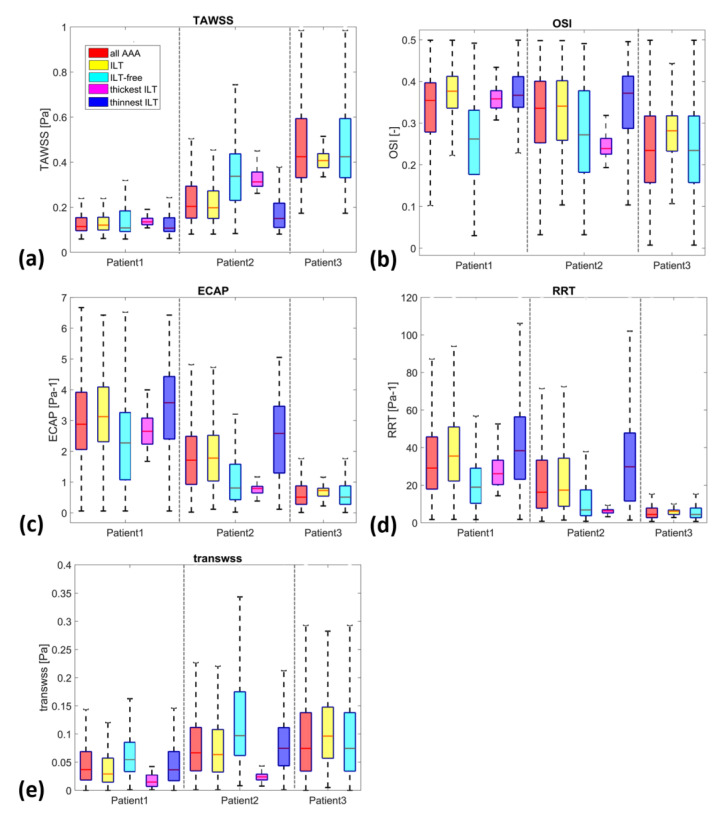
Boxplots of WSS-based parameters (**a**) TAWSS, (**b**) OSI, (**c**) ECAP, (**d**) RRT and (**e**) TransWSS, in the entire AAA, ILT, ILT-free, thickest ILT and thinnest ILT regions for P1, P2 and P3. Whiskers refer to the 10th and the 90th percentiles, respectively; each box ranges between the first and the third quartiles with red line designating the median value of the WSS derivatives.

For a more quantitative comparison, Figure 7 presents boxplots comparing WSS derivatives in the three patients at different regions, including all AAA, ILT, ILT-free, thickest ILT and thinnest ILT regions. The colored boxes indicate the interquartile range (IQR), and the whiskers extend from the box to the minimum and maximum values, excluding outliers. As noted earlier, TAWSS values in patients with ILT (P1 and P2) are lower than in patients with small ILT (P3), as shown in Figure 7a. The OSI values also show differences between P1 and P2 and P3, with a lower IQR and median value in P3. The ECAP and RRT metrics, alongside TAWSS, appear to be the most effective parameters for differentiating between the three patients in terms of the extent of values, their median and the interquartile range.

The yellow and cyan boxplots display the WSS derivatives in the ILT and ILT-free regions, respectively. ILT is associated with low TAWSS for all three patients, with a slight exception for P3. The OSI values in the ILT regions span a broad range of values (>0.21, >0.1 and >0.1 and <0.44 for P1, P2 and P3 respectively). The TransWSS values extend from 0 to 0.12 Pa for P1, to 0.22 Pa for P2 and to 0.28 Pa for P3. The 90th percentiles of ECAP reach 6.4, 4.85 and 1.15 Pa^−1^ and of RRT extend to 95, 74 and 10 Pa^−1^ for patients P1, P2 and P3, respectively. On the other hand, the cyan boxplots representing ILT-free regions cover a larger range of data sets compared to that of ILT regions for all the WSS derivatives and the entire group. Although the IQR differs, the range of values for ILT is included within the range of ILT-free (with the exception of RRT in patient P1 and RRT and ECAP in patient P2). This could be due to the fact that ILT-free regions may encompass areas with hemodynamic conditions that are favorable for ILT accumulation.

The Spearman correlation coefficient (SCC) (see Table 3) was used to analyze the correlations between ILT thickness and WSS derivatives (statistical significance at *p* < 0.001).The results show a significant (*p* < 0.001) but very weak to moderate correlation in the two patients (Patient 1 and Patient 2) (Spearman’s Rho = 0.1951 and 0.4026 for TAWSS, Spearman’s Rho = 0.0671 and −0.1991 for OSI, Spearman’s Rho = −0.2560 and −0.1662 for TransWSS, Spearman’s Rho = −0.1569 and −0.3811 for ECAP, and Spearman’s Rho = −0.0741 and −0.3245 for RRT in Patient 1 and Patient 2, respectively). These results are significant and suggest a relationship between ILT thickness and WSS derivatives, although the strength of the correlation varies.

Motivated by the last observation of the differences between P1 and P2 (P3 was not included in this part of the study since it does not show both thinnest and thickest ILT areas), we investigated whether we can use the hemodynamic characteristics in the thinnest and thickest ILT in AAAs as possible indicators of ILT growth, assuming that the thresholds and magnitude of WSS descriptors found in the literature and used in this study are the most favorable hemodynamic conditions to determine areas of AAA where ILT will continue to grow.

**Figure 8:**
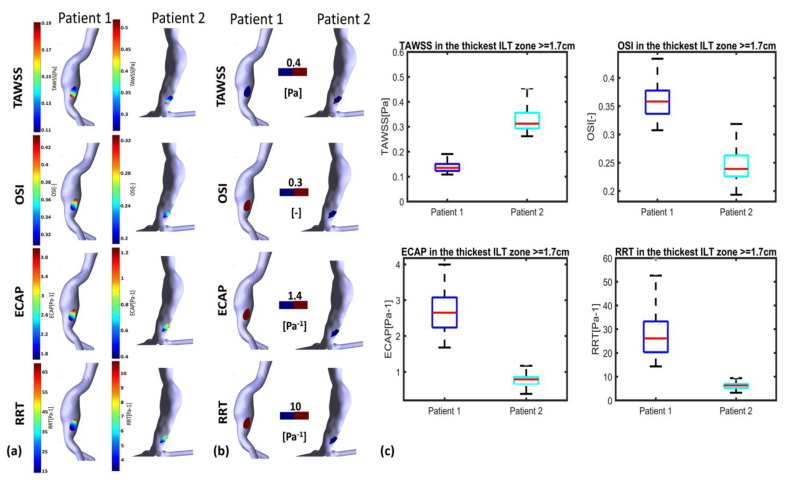
Visualization of TAWSS, OSI, RRT and ECAP in P1 and P2 using CFD simulations in the thickest ILT zones with threshold ≥1.7 cm: (**a**) values indicated in the colormaps; (**b**) values indicated in the colormaps with corresponding threshold used for each WSS-based parameter (blue for values below threshold and red for values above); and (**c**) statistical boxplots.

**Figure 9:**
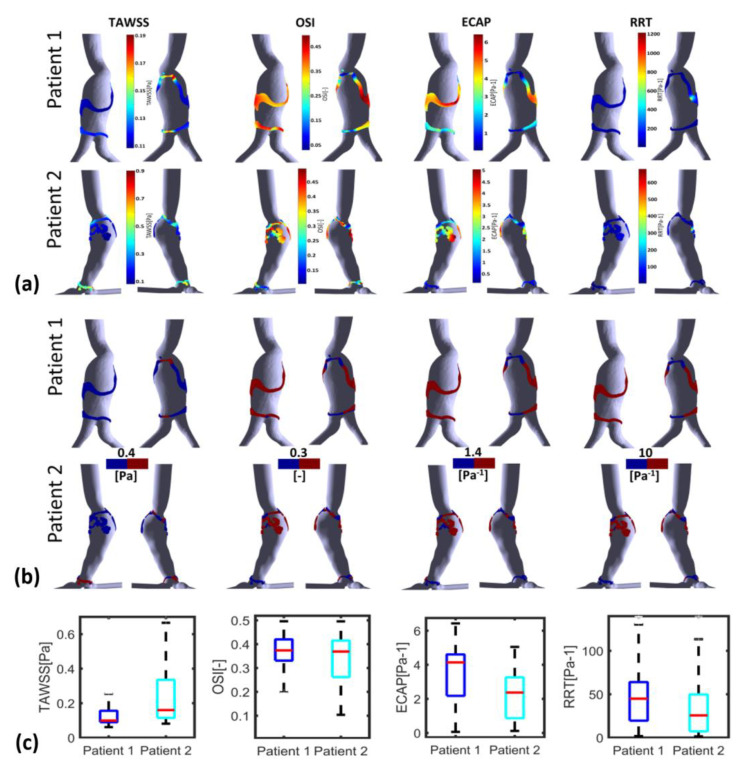
Visualization of TAWSS, OSI, RRT and ECAP in P1 and P2 using CFD simulations in the thinnest ILT zones with threshold ≤0.15 cm: (**a**) values indicated in the colormaps; (**b**) values indicated in the colormaps with corresponding threshold used for each WSS-based parameter (blue for values below threshold and red for values above); and (**c**) statistical boxplots.

Figure 8 and Figure 9 illustrate WSS descriptors for P1 and P2 in the thinnest and thickest ILT areas, respectively. Figure 8a shows TAWSS, OSI, ECAP and RRT contours while Figure 8b shows the hemodynamic descriptors distribution with a threshold to better visualize regions of low and high values. Statistical boxplots are given in Figure 8c. For P1, TAWSS is lower than the threshold value of 0.4 Pa (0.08–0.18 Pa) while OSI, ECAP and RRT are higher than their corresponding threshold values. However, P2 presents with the highest TAWSS compared to P1 at the thickest ILT area, with values ranging between 0.26 Pa and 0.45 Pa. OSI, ECAP and RRT values are lower than the thresholds. The results of thickest ILT regions in this study corresponds to regions of ILT thickness exceeding or equal to 1.7 cm. Comparable trends and observations are found when using 1.5 cm of thickness as the minimum value to define the thickest ILT zone, as well as in thinnest ILT zones where a similar trend was found when 0.3 cm is used as a threshold to define thinnest areas instead of 0.15 cm (data are available upon request for regions with ILT thickness ≥1.5 cm and ≤0.3 cm).

The WSS derivatives in the thinnest ILT regions are presented in Figure 9. The colormaps in Figure 9a reveal that the thinnest ILT regions of P1 exhibit WSS values that are favorable for ILT deposition in the proximal and distal parts of the AAA, with the exception of the posterior wall. Conversely, for P2, a distinct difference in the WSS derivatives values is observed between the proximal and distal parts of the aneurysm. Specifically, the proximal part of the thinnest ILT areas is more favorable for ILT growth compared to the distal part.

The SCCs for P2 show more favorable conditions in the thinnest regions for all the parameters, TAWSS, OSI, ECAP and RRT, where low TAWSS are in the thinnest region compared to the thickest regions; additionally, for OSI, ECAP and RRT, the SCCs are negative which means that the highest values (favorable conditions) are in the thinnest regions. For P1, similarly to P2, SCCs are negative but with lower values compared to P2 (for ECAP and RRT, the SCCs are, respectively, −0.1569 vs. −0.3811 and −0.0741 vs. −0.3245) or show very low values for OSI (0.0671 vs. −0.1991). Furthermore, for TAWSS, SCC in P1 is positive but lower than P2 (0.1951 vs. 0.4026) (see Table 3). These remarks suggest that the ILT growth is more likely to progress in the thinnest areas and especially in the interior proximal wall of the AAA for P2, whereas for P1, the growth of ILT could be predicted in the thickest and thinnest regions uniformly with a slightly stronger potential of growth in the thinnest regions.

It is important to note that this correlation should not be confused with the typical correlation between ILT growth and WSS derivatives. The correlation presented here aims to clarify the distribution of WSS derivatives relative to ILT thickness. This information combined with the WSS derivatives value and range in the thickest and thinnest area of ILT may serve as an additional indicator to understand the ILT growth process, predict regions where ILT is susceptible to continue to grow and estimate regions of the ILT with a strong potential of growth.

## 4. Discussion

This study investigates the relationship between ILT deposition and the hemodynamic parameters related to WSS exerted on the ECs. Three AAA patients with different ILT deposition areas and thicknesses are used for this pilot study to explore the possibility of using the hemodynamic characteristics in the thinnest and thickest ILT areas as possible indicators in managing AAA growth. The co-localization and relationship between WSS-based hemodynamic parameters and ILT deposition are addressed.

The results indicate that ILT accumulates in the recirculation flow area with a low-velocity magnitude. Regions devoid of ILT correlate to jet impingement areas formed inside the aneurysm sac. This agrees with previous findings [12,14]. The jet is formed at the neck and circulated through the AAA and impinges on the anterior wall as seen in P1 and P3 (Figure 4). For these two patients, the anterior wall is free of ILT, whereas the circulating zones created in the surrounding area, mainly in the posterior wall, are characterized by ILT accumulation. This finding endorses the role of vortical structure in ILT accumulation suggested by Zambrano et al. in 2022 [19].

P3 presents a case of ILT initiation near the maximum diameter of the aneurysm lumen. This finding also agreed with the results found by Zambrano et al. [17] showing that the ILT accumulation process begins at regions of maximum diameter. This patient represents a case of small AAA where ILT initiate at regions of high TAWSS and low OSI as it could be seen in boxplots. This observation was reported in Arazani et al. [22] and O’Rourke et al. [23] for small AAA; although, if we see the threshold figure (see Figure 5c), the ILT initiation region is surrounded by regions of low TAWSS and high OSI. However, for P1 and P2, each presented with both thin and thick ILT areas. The maximum ILT thickness is found in the distal part on the left side of the aneurysm (see red zones in the rightmost column of Figure 5a,b). Figure 6 shows the relationship between ILT deposition and the WSS-based hemodynamic parameters. There is a correlation between ILT deposition and low TAWSS values and a direct correlation exists between OSI, RRT and ECAP with ILT. As can be observed, P3 is devoid of ILT and is characterized by higher TAWSS values in the AAA wall and lower OSI, RRT and ECAP values. Conversely, the aneurysm wall in P1 and P2 is almost covered by ILT, corresponding to lower TAWSS and higher OSI, RRT and ECAP values. This agrees with the results found by Tzirakis et al. [54] where patients with significant ILT deposition present low TAWSS values, below 0.5 Pa in the aneurysm sac (in our case, less than 0.22 Pa and 0.51 Pa for P1 and P2, respectively), and for patients devoid of ILT, the TAWSS attain higher values (reaching 1 Pa in P3).

Our current study is one of the fewest exploring the effect of TransWSS on ILT accumulation. Our results indicate that regardless of the nature of the flow near the wall characterized by TransWSS (disturbed flow or non-disturbed flow), the ILT deposition area corresponds to low TAWSS and high OSI values as defined in Table A2, Table A3 and Table A4. However, it was observed that high TransWSS values co-localized with high TAWSS and low OSI. This remark is in accordance with the results found by Lozowy et al. in 2017 in the case of the relationship between OSI and TransWSS [14], whereas low TransWSS values co-localized with low TAWSS and high OSI regions. The opposite of the last two observations is not always valid.

The results indicate that WSS-based hemodynamic parameters are favorable to ILT growth in the thickest and thinnest area for P1. However, for P2, the hemodynamic characteristics are unfavorable to ILT growth in the thickest region and thinnest ILT regions in the distal part of the aneurysm, and favorable conditions are found in the thinnest part situated in the proximal part of the AAA. This suggests a strong potential for ILT growth in the thick ILT area for P1 while P2 shows a weak potential. The possible continuous growth of maximum ILT thickness for P1 can be predicted. This result shows the possibility of predicting ILT growth using the approach of determining the WSS-based indicators in the thinnest and thickest ILT areas of AAA patients. As such, this study has revealed the existence of differences in hemodynamic characteristics in regions of ILT depositions. The identification of these characteristics in the thickest and thinnest ILT areas using non-invasive CFD could help clinicians in the management of the patients. From this conclusion, this study opens the way to a new perspective in the study of the relationship between WSS derivatives and ILT development.

The results of the present study are promising and support the effectiveness of using CFD as a tool that clinicians can use in decision-making regarding the management of patients with AAA. Although this study is limited to three patients, the suggested approach showed interesting results in this group characterized by different ILT deposition areas. Further verification of current results is required, and a replication of this analysis should be performed using a larger sample size of patients, along with the acquisition of a follow-up CT scan to develop a clinical tool for the management of patients presenting with AAA.

## 5. Conclusions

This study focuses on investigating the co-localization and the relationship between ILT and WSS-based hemodynamic parameters in patients with AAA using CFD simulations. Our results indicate that the suggested approach based on the estimation of CFD-based WSS indices in the thin and thick ILT areas extracted from the segmentation of real medical imaging data could provide a tool to support clinicians’ assessment and management of AAA patients. High values of OSI, RRT and ECAP were found to co-localize with ILT deposition areas, whilst there was a correlation between regions with low TAWSS and ILT. ILT deposition areas were found in regions of low TAWSS and high OSI independently of the nature of flow near the wall characterized by TransWSS. This study not only endorsed already accepted hypotheses but also brought some new insights into the management of AAA patients. It can predict where ILT will continue to grow and consequently determine regions susceptible to possible rupture. After the validation using a larger cohort of patients with follow-up data, this approach could be used to score regions of high susceptibility to ILT growth as regions presenting a higher risk of rupture.

## Figures and Tables

**Table 1:** Three AAA patients’ geometrical features and details.

Patient	Age (Years)	Gender	Maximum Lumen AAA Diameter (mm)	Inlet AAA Diameter (mm)	Right Iliac Diameter (mm)	Left Iliac Diameter (mm)	Length of AAA (mm)	Max ILT Thickness (mm)	ILT Thickness/ILT Distribution
P1	70	male	56.7	25.2	15.9	17.2	118.1	19.9	thick-thin/partially in AAA sac
P2	76	male	45.2	23.6	16.5	14.9	146.5	18.5	thick-thin/entire in AAA sac
P3	60	male	32.4	19.6	13.4	12.9	86.3	1.6	thin/small part of AAA sac

**Table 2:** Details of the meshes used in the numerical calculation of AAA patients.

Patient	Number of Cells (Millions)	Number of Nodes (Millions)
P1	3.799	0.948
P2	3.877	0.985
P3	3.885	1.024

**Table 3:** Spearman correlation coefficients between ILT thickness and WSS parameters.

Patient	ILT Region	TAWSS vs. ILT Thickness	OSI vs. ILT Thickness	TransWSS vs. ILT Thickness	ECAP vs. ILT Thickness	RRT vs. ILT Thickness
P1	All ILT	0.1951	0.0671	−0.2560	−0.1569	−0.0741
P2	All ILT	0.4026	−0.1991	−0.1662	−0.3811	−0.3245

All coefficients show *p* values < 0.001.

**Figure A1:**
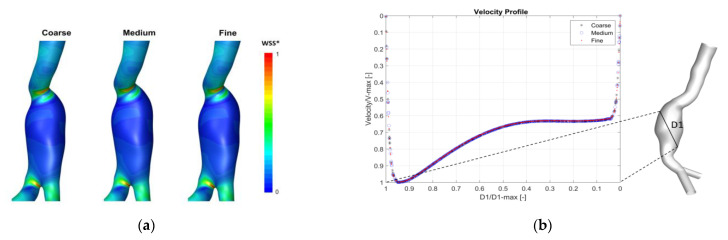
(**a**) Example of qualitative results showing wall shear stress contours in the three meshes for Patient 1 (posterior view is displayed to show the peak values). WSS* is normalized with respect to WSS computed on the fine grid. (**b**) Velocity profile across a line in the center of the aneurysm in Patient1 at systolic peak. Curves are CFD results for three grids (Coarse, Medium and Fine). Velocity values are normalized with respect to V-max in this line.

## Data Availability

The original data are available from the first author and the corresponding author upon an appropriate request.

## Appendix A. Mesh Sensitivity Analysis

To define the optimum computational domain for each AAA model, a mesh sensitivity study was performed by running unsteady flow simulations in ANSYS Fluent using three different meshes (coarse, medium and fine) for each patient, as shown in Table A1. ANSYS Icem CFD (ANSYS, Inc., Canonsburg, PA, USA) was used for the mesh generation. The numerical model and boundary conditions detailed in the methodology section were used (see Section 2). The results at systolic peak were compared between the three meshes. A similar velocity pattern and Wall Shear Stress (WSS) distribution were captured by all three meshes for the three patients, as shown in Figure A1a. The WSS contours for Patient 1 (one patient is shown here as illustration) presented comparable distribution and the highest computed WSS occurred at similar locations, but different maximum values of (WSS_max) were found with the different grids (see Table A1).

**Table A1:** Grid Convergence Index (GCI) and Grid parameters for the 3 AAA patients with corresponding WSS_max values.

	Grid ^1^	Number of Cells (Millions)	WSS_Max (Pa)	GCI (%)
Patient 1	g1 Fine	6.379	4.856	GCI1-2 = 0.102
	g2 Medium	3.799	4.872	GCI2-3 = 0.327
	g3 Coarse	2.306	4.822	
Patient 2	g1 Fine	2.364	25.559	GCI1-2 = 1.448
	g2 Medium	3.877	25.132	GCI2-3 = 0.651
	g3 Coarse	6.351	25.320	
Patient 3	g1 Fine	6.763	18.167	GCI1-2 = 0.035
	g2 Medium	3.885	17.630	GCI2-3 = 0.209
	g3 Coarse	2.236	17.891	

^1^ g1, g2 and g3 refer to grid 1, grid2 and grid3, respectively.

Additionally, a comparison of velocity profile in the three grids with at least two lines inside the aneurysm for each patient was performed. A similar profile with an error <1% was found for the three patients. Figure A1b shows an example of the velocity profile across one line in the center the aneurysm sac of Patient 1.

Furthermore, the mesh sensitivity analysis was endorsed by the calculation of the Grid Convergence Index GCI (Equations (A1)–(A6)), and the maximum WSS (WSS_max) was used as a parameter of interest. The GCI measures the percentage of difference between the computed parameter (WSS_max) for two consecutive grids and estimates how far it is from the asymptotic value. The use of this index is considered as the most reliable technique for the quantification of numerical uncertainty [29]. The mesh sensitivity analysis revealed that the solution became relatively independent to further mesh grid refinement. The medium meshes were used for each patient corresponding to medium-to-fine GCI of approximately 0.327%, 0.651% and 0.209% for Patient 1, Patient 2 and Patient 3, respectively (see Table A1), which is in agreement with recent studies where the grid is adopted if GCI was inferior to 5% [56].

(A1) $${\mathrm{GCI}}_{g1,g2}=F_{s}\cdot \left|E_{g1,g2}\right|$$

(A2) $${\mathrm{GCI}}_{g2,g3}=F_{s}\cdot \left|E_{g2,g3}\right|$$

(A3) $$E_{g1,g2}=\frac{\log\left(\frac{f_{g2}-f_{g1}}{f_{g1}}\right)}{r^{p}-1}$$

(A4) $$E_{g2,g3}=\frac{\log\left(\frac{f_{g3}-f_{g2}}{f_{g2}}\right)}{r^{p}-1}$$

(A5) $$r\approx {\left(\frac{N_{g1}}{N_{g2}}\right)}^{1/3}\approx {\left(\frac{N_{g2}}{N_{g3}}\right)}^{1/3}$$

(A6) $$p=\frac{\log\left(\frac{f_{g3}-f_{g2}}{f_{g2}-f_{g1}}\right)}{\log r}$$

$F_{s}$ is the factor of safety which is equal to 1.25 [56]; $f_{\mathrm{gi}}$ corresponds to the variable of interest (in our case WSS_max); and $N_{\mathrm{gi}}$ is the number of cells in the grid.

## Appendix B. Validation

To validate the numerical model, we performed a comparison of the axial velocity profile at the center of the aneurysm with the experimental and numerical data of Budwig et al. [37] for steady flow through axisymmetric AAA model. The simulations are carried out at Re = 2000 and Re = 400, aneurysm-to-artery diameter ratio D/d = 2.1 and an aspect ratio of the aneurysm L/d = 4 (L is the length of the aneurysm). The flow feature is characterized by a jet formation surrounded by recirculating vortex, and an agreement was found between the present results and the description given by Budwig et al. [37]. The velocity profiles across the aneurysm are in good agreement with the experimental and numerical results of Budwig et al. [37] (see Figure A2a and Figure A2b, respectively), but the shape of the aneurysm was not detailed in this reference. The possible difference of the shape of the aneurysm may be the cause of the slight difference in the velocity profile compared to the provided experimental and numerical results.

## Appendix C

For the three patients, the AAA sac is characterized by low TAWSS values (high InvTAWSS) compared to the healthy part of the aorta. The maximum values of InvTAWSS (min values of TAWSS) are found in the dome of the aneurysm reaching values of maximum InvTAWSS superior to 17, 12 and 6 Pa^−1^ for P1, P2 and P3, respectively. From these results, it can be observed that low TAWSS (high InvTAWSS) are linked with ILT deposition regions. Patient 3 presents a higher range of TAWSS values (lower range of InvTAWSS) in the AAA sac compared to P1 and P2. This patient is characterized by small and dispersed ILT deposition areas in the aneurysm sac.

For P2, high values of TransWSS are observed in the two iliac arteries and in some dispersed areas of the AAA sac, whereas an inverse relationship was found between TransWSS and OSI in the entrance of AAA sac for P1, where high values of TransWSS coincide with low OSI in this area, and the wall is devoid of ILT deposition. Conversely, the wall is devoid of ILT in some areas for P2 with low values of both OSI and TransWSS.

In the aneurysm sac for P1 and P2, the ILT deposition is correlated with high oscillating flow corresponding to high OSI values, high RRT of blood particles near the wall and low TAWSS (high InvTAWSS) values.

## Appendix D

**Table A2:** ILT deposition regarding flow near the wall to which endothelial cells (ECs) are subjected to in region of interest (AAA sac) for Patient 1 (P1).

	AAA Areas	WSS Parameters			Flow Near the Wall to Which ECs Are Subjected to *	ILT Deposition
		TAWSS	OSI	TransWSS		
P1	Entrance of AAA	-High	-Low	-High	-Unidirectional disturbed flow	-No ILT
	AAA Sac	-Low	-Low -High	-High and (low in some regions) -Low -Proportionally High in some regions	-Unidirectional disturbed flow (non-disturbed flow) -Oscillating non disturbed flow - Oscillating disturbed flow	-No ILT -ILT
	Downstream AAA sac	-High	-Low	-High	-Unidirectional disturbed flow	-No ILT

* Graphical representations of flow near the wall to which ECs are subjected to.

**Table A3:** ILT deposition regarding flow near the wall to which endothelial Cells (ECs) are subjected to in region of interest (AAA sac) for Patient 2 (P2).

	AAA Areas	WSS Parameters			Flow Near the Wall to Which ECs Are Subjected to *	ILT Deposition
		TAWSS	OSI	TransWSS		
P2	Entrance of AAA	-High	-Low	-Low -Proportionally High in some regions	-Unidirectional non disturbed flow -Unidirectional disturbed flow	-No ILT
	AAA Sac	-Low	-High	-Low -Proportionally High in some regions	-Oscillating non disturbed flow - Oscillating disturbed flow	-ILT
	Downstream AAA sac	-High	-Low	-High	-Unidirectional disturbed flow	-No ILT

* Graphical representations of flow near the wall to which Endothelial cells EC are subjected to.

**Table A4:** ILT deposition regarding flow near the wall to which endothelial cells (ECs) are subjected to in region of interest (AAA sac) for Patient 3 (P3).

	AAA Areas	WSS Parameters			Flow Near the Wall to Which ECs Are Subjected to *	ILT Deposition
		TAWSS	OSI	TransWSS		
P3	Entrance of AAA	-High -Low	-Anterior part low -Posterior part high	-High	-Unidirectional disturbed flow -Oscillating disturbed flow	-No ILT (anterior) A -ILT (posterior) P
	AAA Sac	-Low -High	-High Dmax zone (belt from dome to posterior) -Low in the remaining regions	-Low	-Oscillating non disturbed flow -Unidirectional non disturbed flow	-ILT -No ILT
	Downstream AAA Sac	-High	-Low	-Low	-Unidirectional disturbed flow	-No ILT

* Graphical representations of flow near the wall to which Endothelial cells EC are subjected to.
